# Comparative Time-Course Physiological Responses and Proteomic Analysis of Melatonin Priming on Promoting Germination in Aged Oat (*Avena sativa* L.) Seeds

**DOI:** 10.3390/ijms22020811

**Published:** 2021-01-15

**Authors:** Huifang Yan, Peisheng Mao

**Affiliations:** 1 Forage Seed Laboratory, China Agricultural University, Beijing 100193, China; yanhuifang@qau.edu.cn; 2 Grassland Agri-Husbandry Research Center, College of Grassland Science, Qingdao Agricultural University, Qingdao 266109, China

**Keywords:** melatonin, time course of priming, seed aging, germination, oat, proteomics

## Abstract

Melatonin priming is an effective strategy to improve the germination of aged oat (*Avena sativa* L.) seeds, but the mechanism involved in its time-course responses has remained largely unknown. In the present study, the phenotypic differences, ultrastructural changes, physiological characteristics, and proteomic profiles were examined in aged and melatonin-primed seed (with 10 μM melatonin treatment for 12, 24, and 36 h). Thus, 36 h priming (T36) had a better remediation effect on aged seeds, reflecting in the improved germinability and seedlings, relatively intact cell ultrastructures, and enhanced antioxidant capacity. Proteomic analysis revealed 201 differentially abundant proteins between aged and T36 seeds, of which 96 were up-accumulated. In melatonin-primed seeds, the restoration of membrane integrity by improved antioxidant capacity, which was affected by the stimulation of jasmonic acid synthesis via up-accumulation of 12-oxo-phytodienoic acid reductase, might be a candidate mechanism. Moreover, the relatively intact ultrastructures enabled amino acid metabolism and phenylpropanoid biosynthesis, which were closely associated with energy generation through intermediates of pyruvate, phosphoenolpyruvate, fumarate, and α-ketoglutarate, thus providing energy, active amino acids, and secondary metabolites necessary for germination improvement of aged seeds. These findings clarify the time-course related pathways associated with melatonin priming on promoting the germination of aged oat seeds.

## 1. Introduction

Oat (*Avena sativa* L.) is an important annual herbaceous crop in the family Gramineae. It is also an extremely nutritious cereal that possesses high levels of protein, soluble dietary fiber, and minerals [1]. Oat is widely utilized as both a food for human consumption and a hay product for animal husbandry [2]. However, its seeds deteriorate easily with aging, ascribing to the high oil content and resulting in the oxidation rancidity of its polyunsaturated fatty acids [3], which directly limits its extensive use and causes great economic losses.

Seed aging, which leads to a loss of vigor, is a serious problem for natural storage and utilization in agriculture. The decreased vigor further gives rise to poor and non-uniform seed germination, which subsequently influences seedling performances under multifarious conditions [4]. Accumulation of reactive oxygen species (ROS) is considered to be a primary reason of seed aging, causing various harmful metabolic changes, such as the oxidation of lipids and proteins, degradation of DNA and RNA, and disruption of membrane integrity [5]. Our previous physiological and biochemical studies focusing on oat seeds revealed that artificially aging at 45 °C led to severe damage to mitochondrial integrity [6] and lower levels of activity in the antioxidant scavenging system [7]. Moreover, proteomic analysis also uncovered that various differentially abundant proteins (DAPs) in oat seed embryos were induced by aging, mainly involved in carbohydrate and energy metabolism, protein translation, and amino acid metabolism [8,9]. For aged oat seeds, delayed germination and repressed post-germination growth are the two biggest impediments that restrict its broader application. As the most important initial stage of crop development and growth, germination of seeds with low vigor requires improvement and is a major challenge in field planting, especially under stressful environments. Therefore, it is essential to develop strategies to rejuvenate the vigor of aged seeds, in order to stimulate their germination.

Seed priming can reinforce the vigor of aged seeds and enhance their germination, by efficaciously triggering metabolic events before radicle protrusion [10]. The mechanism through which priming treatments improve seed viability is related to the repair of cellular and mitochondrial components [11], synthesis of nucleic acids and proteins, and recovery of antioxidants [12]. Previous studies indicated that priming with exogenous substances, e.g., ascorbic acid (AsA), glutathione (GSH), and salicylic acid (SA), significantly accelerated the germination of aged seeds in many species, including Siberian wildrye (*Elymus sibiricus* L.) [11], oat [13], and soybean (*Glycine max* (L.) Merr.) [14]. Although the involved structural and physiological changes have been explored [15,16], the proteomic differences and molecular mechanisms underlying the priming effects that promote germination of aged seeds are still relatively limited.

Melatonin (*N*-acetyl-5-methoxytryptamine) is a natural and non-toxic molecule that has multiple functions and occurs in diverse organisms including plants [17]. As a strong and effective endogenous antioxidant, it was proved to accelerate seed germination [18], delay leaf senescence [19], and ameliorate damage caused by chilling, drought, and salinity [20,21,22]. Melatonin was reported to function through scavenging excess ROS directly [23], and enhancing mitochondrial efficiency, antioxidant capacity and the expression of related genes indirectly under adverse conditions [24,25]. Hitherto, studies that have focused on melatonin’s role in regulating the germination of aged seeds are still very rare [26]. Our previously published results suggested that melatonin priming had great potential in alleviating aging-induced germination inhibition in oat seeds, relying on its concentration variation, by regulating β-oxidation, protein translation, and antioxidant metabolism [9]. In addition, the effects of seed priming are also time-course dependent; however, the related pathways and mechanisms of melatonin expediting the germination of aged oat seeds based on different priming durations are unknown.

Therefore, this study was conducted to determine the changes in ultrastructural repair, remission of hydrogen peroxide (H_2_O_2_) accumulation and lipid peroxidation, improvement of antioxidant scavenging capacity, and DAP accumulation profiles in embryos between melatonin-primed and aged seeds. The objectives were to investigate whether melatonin priming could promote germination of aged seeds in a time-course dependent manner, and to illuminate whether such an improvement in germination was related to cellular structural recovery, antioxidant capacity increases, and/or substance metabolism and activation.

## 2. Results

### 2.1. Germinability and Seedling Performance of Aged Oat Seeds Following Melatonin Priming

Based on the screening tests for melatonin concentration (Figure S2), it was shown that germination percentage (GP) did not increase after 0.1~1000 μM of melatonin priming for 12 h (T12), and it was only improved significantly by 0.1 μM of melatonin, to a value of 81% after priming for 24 h (T24). Additionally, although all concentrations of melatonin (0.1~1000 μM) significantly improved GP after 36 h of priming (T36), the maximum value (86%) was obtained with 10 μM of melatonin. Therefore, under comprehensive comparison, 10 μM of melatonin was more appropriate for improving GP of aged seeds, and was selected for further experiments to study its time-course related responses (Figure S1).

**Figure 1:**
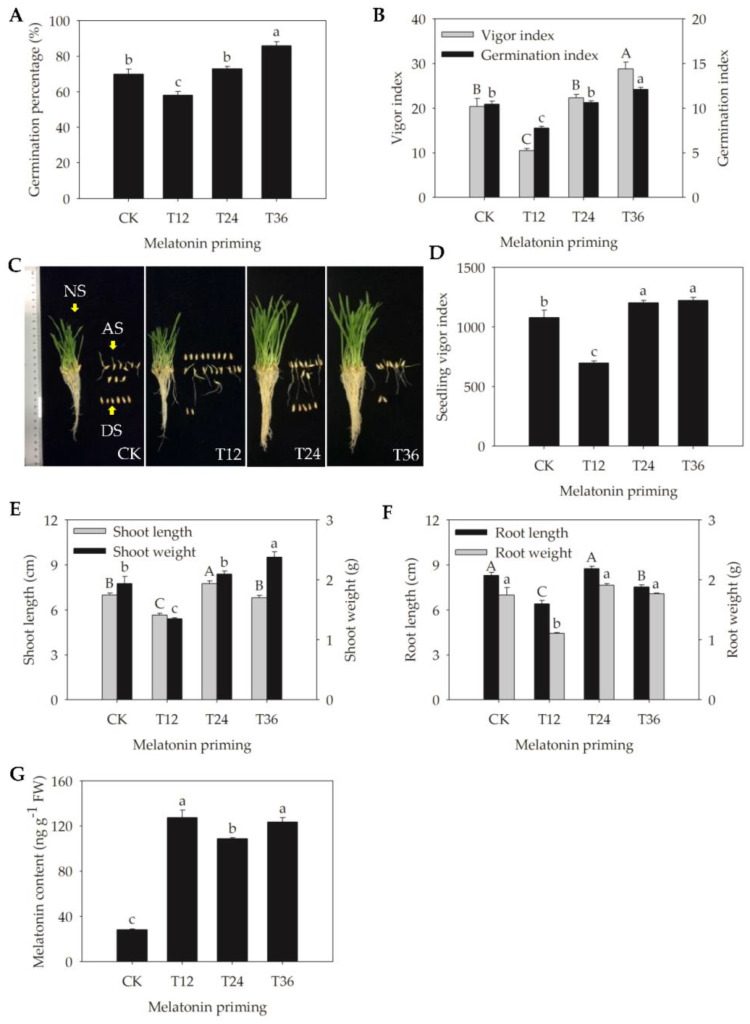
Seed germinability and seedling growth status of aged oat seeds after melatonin priming. (**A**) Germination percentage (GP) (CK, aged seeds; T12~T36, seeds that aged and then were further primed with 10 μM of melatonin for various durations of 12, 24, and 36 h); (**B**) Seed vigor index (VI) and germination index (GI); (**C**) Seedling phenotypic characteristics on the 10th day of germination; yellow arrows showed normal seedlings (NS), abnormal seedlings (AS), and dead seeds (DS), respectively; (**D**) Seedling vigor index (SVI); (**E**) Shoot length (SL) and shoot weight (SW); (**F**) Root length (RL) and root weight (RW) of seedlings; and (**G**) Melatonin content. Values represent the means ± SE from four replicates. One-way ANOVA was adopted to perform the statistical analysis. Different letters indicated significant differences between melatonin-primed seeds and aged seeds at the 0.05 level.

The effects of melatonin on germination and seedling growth differed among various priming durations (Figure 1). As revealed by changes of GP, seed vigor index (VI), and germination index (GI), 10 μM of melatonin priming for both 12 and 24 h could not significantly increase the germinability of aged seeds—T12 especially further exacerbated its decline (Figure 1A,B). Compared to aged seeds (CK), only a priming duration of 36 h significantly enhanced GP, VI, and GI by 16%, 41.5%, and 15.8%, respectively (Figure 1A,B).

Furthermore, the phenotypic performances of normal seedlings were obviously improved and similar after T24 and T36 melatonin priming, but again, only T36 priming had a superior effect because of the lower quantity of abnormal seedlings and dead seeds under this treatment (Figure 1C). These phenotypic results were consistent with changes of seedling vigor index (SVI), shoot length (SL), and shoot weight (SW). Even though both T24 and T36 significantly enhanced SVI (by 11.3% and 13.2%, respectively), they made greater contributions to the increases in SL and SW (by 10.7% and 22.7%, respectively) (Figure 1D,E). However, melatonin priming for various durations actually had no significant positive effect on root length (RL) and root weight (RW) (Figure 1F). Thus, based on the above findings, T36 better improved the germinability and seedling growth of aged oat seeds. Additionally, endogenous melatonin contents of T12, T24 and T36 treatments were all significantly higher than that in aged seeds (Figure 1G).

### 2.2. Ultrastructural Charicteristics of Embryos from Aged Oat Seeds Following Melatonin Priming

**Figure 2:**
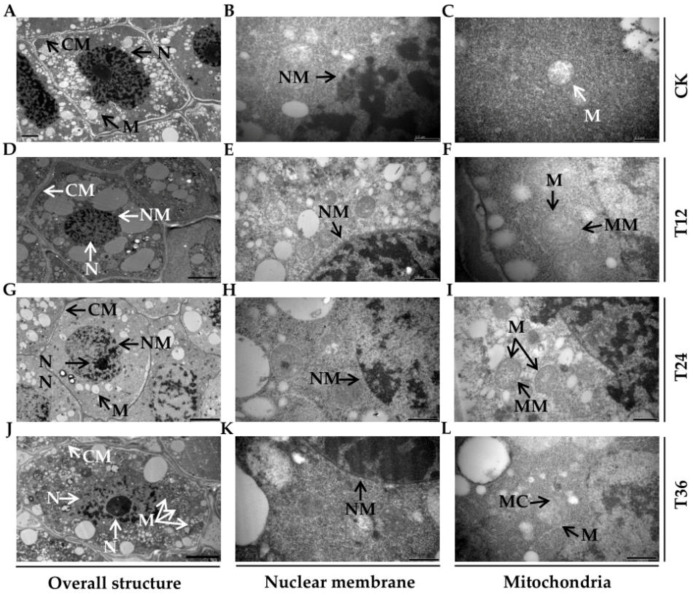
Differences in ultrastructural characteristics of aged oat seeds after melatonin priming. (**A**–**C**) CK. (**D**–**F**) T12. (**G**–**I**) T24. (**J**–**L**) T36. (**A**,**D**,**G**,**J**) Overall structure of embryonic cell; (**B**,**E**,**H**,**K**) Alterations of nuclear membrane; and (**C,F,I,L**) Mitochondrial ultrastructure. CM, cell membrane; N, nuclear; NN, nuclear nucleolus; NM, nuclear membrane; M, mitochondrion; MC, mitochondrial cristae; MM, mitochondrial membrane. Bars = 200 nm (**F**), 0.5 µm (**A**–**C**,**E**,**H**,**K**), and 5 µm (**D**,**G**,**I**,**J**,**L**).

Ultrastructural observation of radicle cells showed that the recovery effect of melatonin on cellular damage was closely related to its priming duration (Figure 2). All T12, T24, and T36 treatments, to a certain extent, restored the following flaws caused by aging: incomplete cytoplasmic membranes and plasmolysis, blurred boundaries between the nucleus and cytoplasm, and vacuolar mitochondria (Figure 2A,D,G,J). However, more nuanced observation revealed that the nucleoli were larger and more clearly visible, and the nuclear membranes were complete, with double layers after the T36 melatonin priming (Figure 2J,K); at the same time, mitochondria also returned to their typically spherical appearances, displaying internally narrow crista and complete double-layer membranes (Figure 2L).

### 2.3. Changes of H_2_O_2_ Accumulation, Lipid Peroxidation, and Antioxidant Capacity in Aged Oat Seeds Following Melatonin Priming

**Figure 3:**
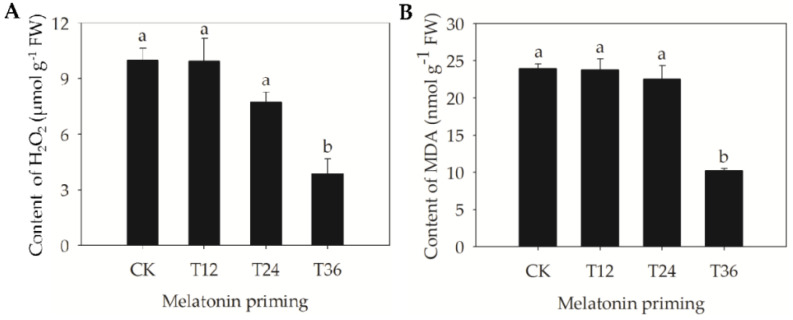
Hydrogen peroxide (H_2_O_2_) accumulation and lipid peroxidation changes in aged oat seeds after melatonin priming. (**A**) H_2_O_2_ content; and (**B**) Malondialdehyde (MDA) content. Values represent the means ± SE from four replicates. One-way ANOVA was adopted to perform the statistical analysis. Different letters indicated significant differences between melatonin-primed seeds and aged seeds at the 0.05 level.

To further evaluate whether the remediation effect of melatonin on aging-caused damage was related to priming duration, physiological responses were also studied. As the results showed, only T36 significantly decreased both the H_2_O_2_ accumulation and malondialdehyde (MDA) content of aged seeds, by 61.3% and 57.2%, respectively (Figure 3). However, there were no significant differences in both H_2_O_2_ and MDA contents among the T24, T12, and CK treatments.

**Figure 4:**
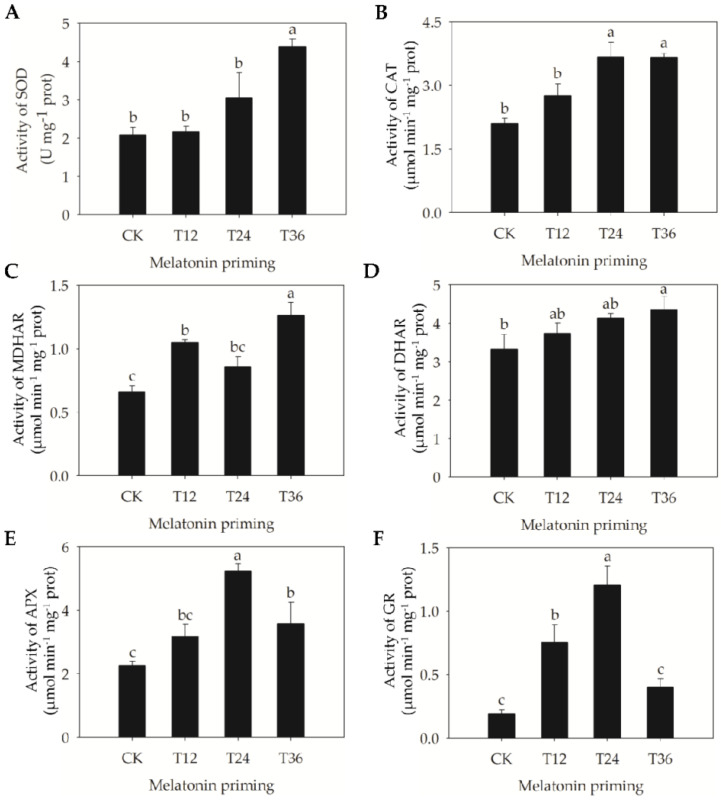
Changes of antioxidant enzymes’ activities in aged oat seeds after melatonin priming. (**A**) Superoxide dismutase (SOD); (**B**) Catalase (CAT); (**C**) Monodehydroascorbate reductase (MDHAR); (**D**) Dehydroascorbate reductase (DHAR); (**E**) Ascorbate peroxidase (APX); and (**F**) Glutathione reductase (GR). Values represent the means ± SE from four replicates. One-way ANOVA was adopted to perform the statistical analysis. Different letters indicated significant differences between melatonin-primed seeds and aged seeds at the 0.05 level.

In addition, the effects of melatonin on activities of different antioxidant enzymes were also strongly linked with priming duration (Figure 4). The activities of monodehydroascorbate reductase (MDHAR) and glutathione reductase (GR) in T12 seeds were significantly higher than those in aged seeds (Figure 4C,F). When priming duration was extended to 24 h, the activities of catalase (CAT) and ascorbate peroxidase (APX) began to significantly increase, by 74.9% and 130.9%, respectively (Figure 4B,E); simultaneously, GR activity also significantly increased, by 521.4% (Figure 4F), with the activities of APX and GR reaching their maximum levels (Figure 4E,F). Furthermore, as priming duration continued to 36 h, superoxide dismutase (SOD) and dehydroascorbate reductase (DHAR) started to be induced and maximized by melatonin, and their activities were increased by 110.9% and 31.2%, respectively (Figure 4A,D); meanwhile, the activities of CAT, MDHAR, and APX were significantly increased, by 74.3%, 91.0%, and 58.0%, respectively (Figure 4B,C,E,F). The above results suggested that although MDHAR and GR responded more sensitively to melatonin at early-stage priming (T12), the activities of more antioxidant enzymes were activated by late-stage priming (T36).

### 2.4. Quantitative Proteomic Analysis Profiles of Oat Seed Embryos Following Melatonin Priming

To investigate the proteins associated with the response to melatonin in aged seeds, the iTRAQ method was performed, using embryos from melatonin-primed seeds (T12, T24, and T36) and aged seeds (CK). A total of 52,054 spectra, 8366 peptides, and 3373 proteins were identified (Table S1).

**Figure 5:**
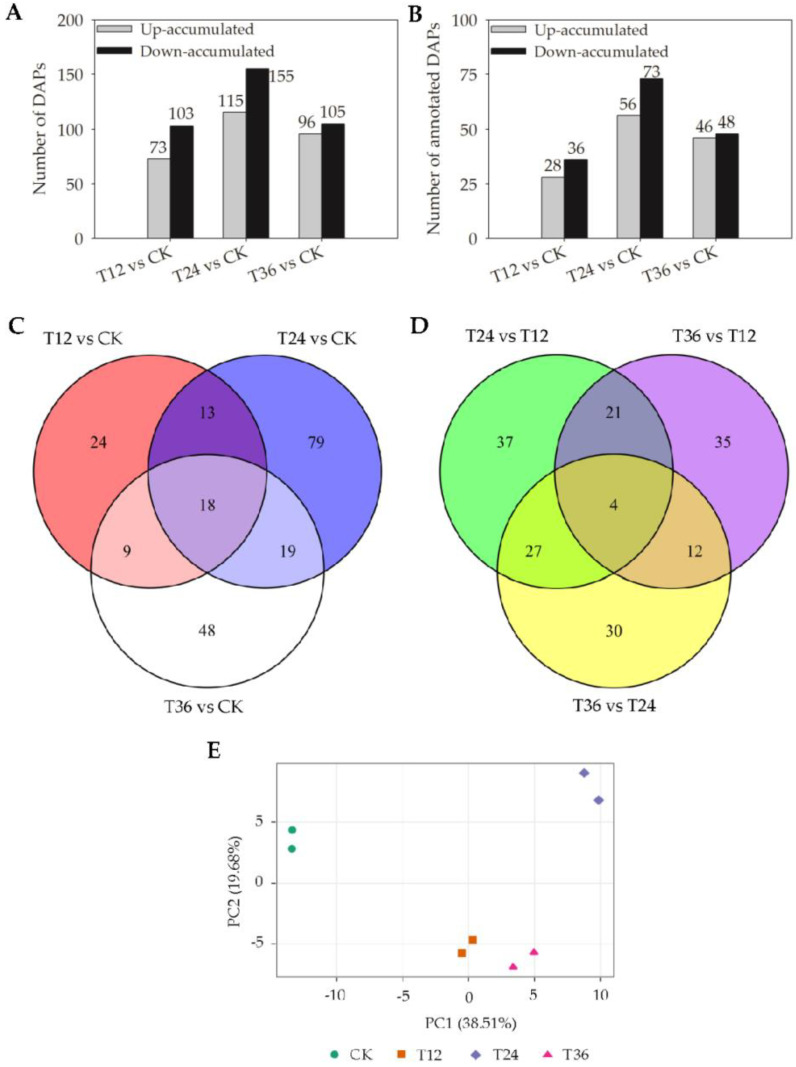
Number of differentially abundant proteins (DAPs) quantified in embryos of melatonin-primed seeds and aged seeds. (**A**) All DAPs between melatonin treatments and control; (**B**) Annotated functional DAPs between melatonin treatments and control; (**C**) Venn diagram of annotated functional DAPs comparing melatonin treatments and control; (**D**) Venn diagram of annotated functional DAPs comparing individual melatonin treatments; and (**E**) PCA analysis of annotated DAPs’ abundance of melatonin-primed seeds (T12, T24, and T36) and aged seeds (CK).

Three pairwise comparisons of proteomes were made to identify DAPs between melatonin-primed treatments and the control. Proteins with a fold change (FC) ≥ 1.5 (or ≤0.67) and *p*-value ≤ 0.05 were considered significantly up-accumulated (or down-accumulated). Based on these criteria, 475 DAPs were identified after melatonin priming (Table S2A). There were 73 up- and 103 down-accumulated DAPs identified in the T12 vs. CK, 115 up- and 155 down-accumulated DAPs identified in the T24 vs. CK, and 96 up- and 105 down-accumulated DAPs identified in the T36 vs. CK, respectively (Figure 5A).

In total, only 210 DAPs were annotated with functions, comprising less than half of all DAPs (Table S2B). In the T12 vs. CK, 64 DAPs were identified, with 28 up- and 36 down-accumulated DAPs; in the T24 vs. CK, 129 DAPs were identified, with 56 up- and 73 down-accumulated DAPs; and in the T36 vs. CK, 94 DAPs were identified, with 46 up- and 48 down-accumulated DAPs, respectively (Figure 5B). Furthermore, among the 210 annotated DAPs, there were 24, 79, and 48 DAPs identified only in the T12 vs. CK, T24 vs. CK, and T36 vs. CK, respectively; 13, 9, and 19 DAPs were shared between the T12 vs. CK and T24 vs. CK, the T12 vs. CK and T36 vs. CK, and the T24 vs. CK and T36 vs. CK, respectively; 18 DAPs were simultaneously overlapped among all three relevant comparisons, i.e., the T12 vs. CK, T24 vs. CK, and T36 vs. CK (Figure 5C). Principal components analysis (PCA) showed that annotated DAP profiles of melatonin-primed seeds differed from those of aged seeds, but it presented a higher similarity between T12 and T36 (Figure 5E), of which the possible reasons were the insufficient repair at a short period of priming (T12) and the best improvement effect at T36 treatment. In addition, annotated functional DAPs comparing individual melatonin treatments were analyzed, showing that there were only four DAPs overlapped by the T24 vs. T12, T36 vs. T12, and T36 vs. T24 (Figure 5D).

### 2.5. Functional Classification Analysis of DAPs in Oat Seed Embryos Following Melatonin Priming

**Figure 6:**
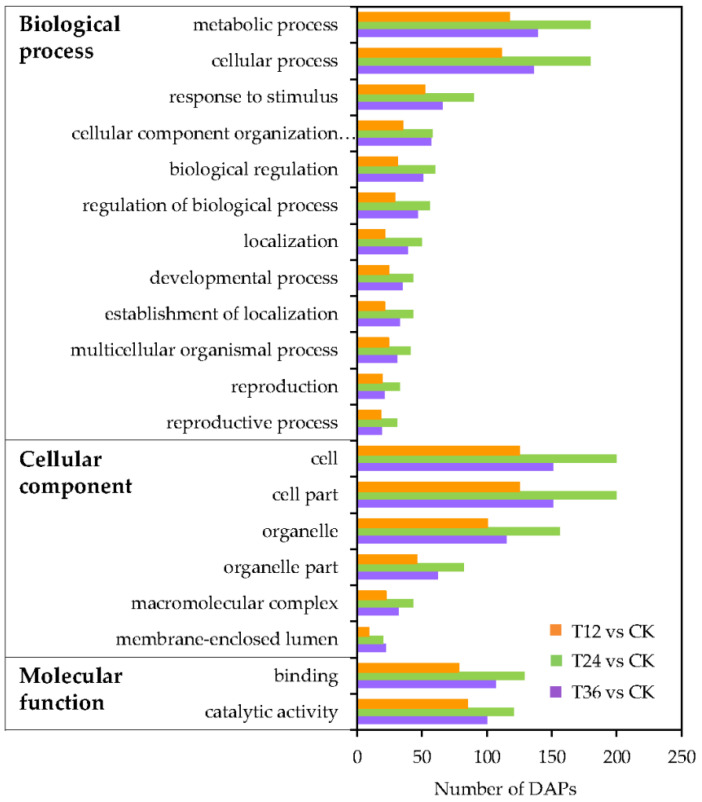
Top 20 gene ontology (GO) categories of all DAPs in embryos of aged seeds after melatonin priming for various durations.

To gain more insights into the response of DAPs to melatonin in aged seeds, all DAPs were subjected to functional classification. Gene ontology (GO) annotations were used to clarify the functional distribution of DAPs after melatonin priming, with the top 20 functional groups identified. Among these, biological process accounted for 12 GO terms, mainly involving metabolic and cellular processes; cellular component accounted for six GO terms, with enrichment of the cell, cell part, and organelle; and molecular function accounted for two GO terms, including binding and catalytic activity (Figure 6).

**Figure 7:**
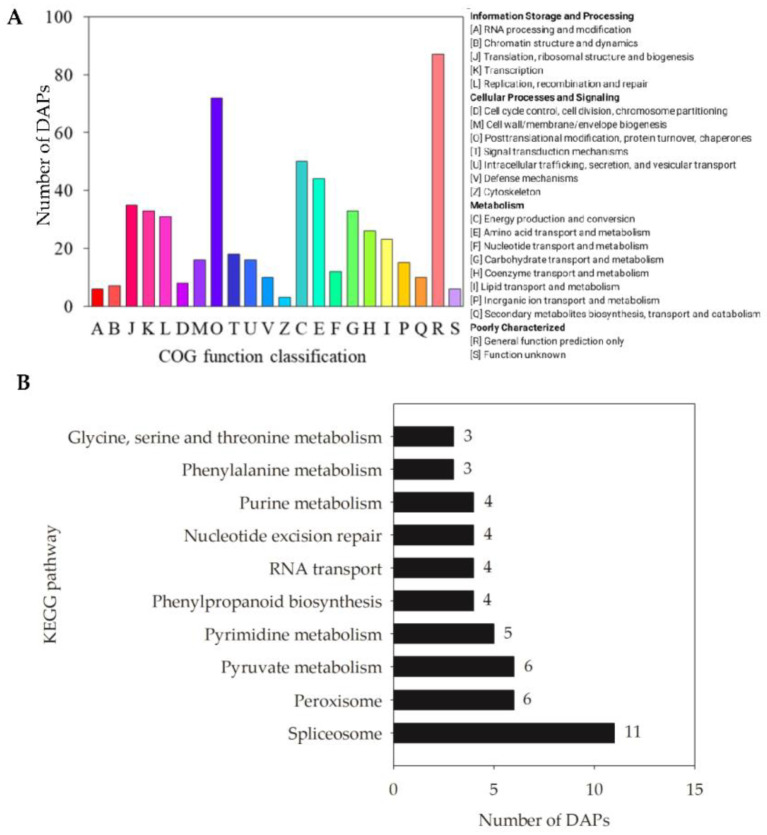
Function classification of all DAPs identified in embryos of aged oat seeds after melatonin priming for various durations. (**A**) Cluster of orthologous groups (COG) function annotation; and (**B**) Top 10 of the Kyoto Encyclopedia of Genes and Genomes (KEGG) function classification, based on those DAPs with at least 2 peptides (including at least 1 unique peptide).

Among the 475 DAPs, 302 were classified into four groups, including 22 categories, according to the Cluster of Orthologous Groups (COG) database (Figure 7A). The DAPs were mainly involved in post-translational modification, protein turnover, and chaperones (category O, 12.8%); energy production and conversion (category C, 8.9%); amino acid transport and metabolism (category E, 7.8%); translation, ribosomal structure and biogenesis (category J, 6.2%); transcription (category K, 5.9%); carbohydrate transport and metabolism (category G, 5.9%); and replication, recombination, and repair (category L, 5.5%). Detailed COG annotations of DAPs classified into different categories are listed in Table S3.

To further explore the biological functions of DAPs in embryos of aged seeds after melatonin priming, pathway analysis was also performed based on the Kyoto Encyclopedia of Genes and Genomes (KEGG) database. Among the top 10 KEGG pathways (Figure 7B), DAPs enriched for involvement in the spliceosome (22%) occupied the largest proportion, followed by the peroxisome (12%) and pyruvate metabolism (12%) pathways. The phenylalanine metabolism and phenylpropanoid biosynthesis pathways involved in phenylalanine and phenylpropanoid accumulation were enriched in melatonin-primed seeds. Additionally, a certain number of DAPs related to amino acid metabolism (e.g., purine, glycine, serine, etc.) were also enriched, indicating that the improved antioxidant ability, enhanced energy production, and activated amino acids by melatonin priming were needed for the germination of aged seeds. Furthermore, one key DAP each in the phosphatidylinositol signaling pathway and the alpha-linolenic acid metabolism pathway was identified in aged seeds after melatonin priming (Table 1).

### 2.6. Confirmation of DAPs by qRT-PCR at the Transcriptional Level

**Figure 8:**
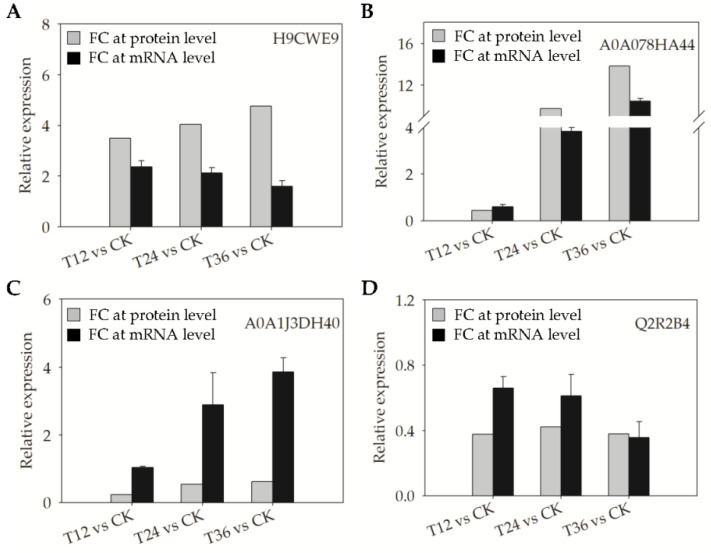
Comparison of expression changes of selected DAPs at the protein and mRNA level in the embryos of aged oat seeds after melatonin priming. (**A**) H9CWE9; (**B**) A0A078HA44; (**C**) A0A1J3DH40; and (**D**) Q2R2B4.

To validate the reliability of proteomic data determined by iTRAQ, qRT-PCR was used to assess DAPs’ expressions at the mRNA transcriptional level. Four DAPs were selected, which accumulated simultaneously in melatonin-primed seeds and aged seeds. With the exception of A0A1J3DH40, the other three DAPs (H9CWE9, A0A078HA44, and Q2R2B4) showed consistent expression trends between the protein and mRNA levels (Figure 8).

## 3. Discussion

Seed aging, which negatively affects both external morphological structures and internal physiological characteristics of seeds, is inevitable and irreversible during storage [27]. It can result in delayed germination, abnormal seedlings, and even a total loss of seed vigor, which in turn causes substantial economic losses and reduced genetic diversity, and it has become a serious problem in agriculture [8]. Seed priming with melatonin is increasingly favored to accelerate germination and/or promote seedling growth (e.g., coleoptile length, fresh weight, and dry weight) in some species, such as cotton (*Gossypium hirsutum* L.) [18], maize (*Zea mays* L.) [20], basil (*Ocimum basilicum* L.) [28], and wheat (*Triticum aestivum* L.) [29], thus enabling them to cope with chilling, aging, salt, and osmotic stresses [20,26,28]. The present study shows that a melatonin priming duration of 36 h significantly counteracted the detrimental effects of aging on oat seeds, as indicated in the improved GP, VI, and GI seedling phenotypes, SVI, and SW (Figure 1). Additionally, melatonin was reported to ameliorate ultrastructural damage of meristematic cells, especially plastids, in chilled and re-warmed mung bean (*Vigna radiata* L.) roots [30]. Likewise, this study also revealed that melatonin repaired structural damage that had already occurred during seed aging, with the T36 priming working best in particular, resulting in larger nucleoli, more complete nuclear membranes, and typically spherical mitochondria (Figure 2J–L). Furthermore, the results of endogenous melatonin content suggested that the concentration of 10 μM was sufficient for aged seeds, because its content was already significantly increased at T12 treatment, and maintained at similar levels among all T12, T24 and T36 treatments (108.62~127.33 ng/g, Figure 1G). However, germination phenotype and cell structures were not improved at T12, which might be due to the secondary damage caused by rapid water absorption at the start of imbibition in dry aged seeds, and priming duration of 12 h was not enough to repair this destruction. Bailly [31] proposed that lipid autoxidation generated primary free radicals during prolonged seed storage, and the resumption of metabolism caused new (secondary) free radicals during germination. A short period of insufficient repair (T12) could not eliminate the old and new damage to macromolecules and cell structures, thus presenting the poor germination.

Previous studies have also suggested that priming with exogenous antioxidants can trigger repair- and germination-related events in aged seeds, mainly via the repair of DNA damage, ATP production, protein synthesis, and activation of some ROS scavenging enzymes [12]. Based on changes in the enzymatic activities of oat seeds, MDHAR and GR responded quickly to melatonin priming at the early stage (T12), followed by CAT and APX at the intermediate stage (T24) (Figure 4B,C,E,F). However, T36 priming was able to activate most antioxidant enzymes, including SOD, DHAR, CAT, MDHAR, and APX, and the activities of SOD and DHAR reached their maximum levels (Figure 4A,D). These results indicated that the early and medium phases of melatonin priming gradually stimulated these enzymes and provided the basis for subsequent resistance to depression in antioxidant capacity of the AsA–GSH cycle. In addition, T36 priming showed a better and stronger improvement effect, and it was therefore beneficial to remove H_2_O_2_. Consequently, H_2_O_2_ and MDA contents in T36 seeds were also significantly lower (Figure 3), which suggested that lipid peroxidation was effectively alleviated. Similarly, Sharma et al. [32] has reported that high activity levels of ROS scavenging enzymes are closely coordinated with the improvement of mitochondrial DNA integrity in primed seeds, which contributes to induce early metabolic activities, thereby promoting the rapid and high germination of aged seeds. Accordingly, the exact consistency of germinative, structural, and physiological findings revealed that restoration of cellular structure and a highly active enzyme system were closely related to the improved germinability of aged oat seeds.

Seed priming is extensively used to improve the vigor and germination of aged seeds [10]. As an effective strategy, proteomic analysis has been demonstrated to create an avenue to reveal molecular mechanisms induced by stimuli, particularly responding to seed aging and priming [33]. To further uncover the underlying embryonic protein changes and to obtain a clearer understanding of the time-course dependent improvement effects of melatonin on germination, an integrated iTRAQ-based proteomic analysis was conducted on aged and primed seeds. In this study, some DAPs were possibly related to the germination of melatonin-primed seeds, which were mainly involved in carbon metabolism and energy production, amino acid metabolism, phenylpropanoid biosynthesis, phosphatidylinositol signaling, and alpha-LINOLENIC acid metabolism, according to KEGG pathway analysis (Table 1).

### 3.1. DAPs Involved in Carbon Metabolism and Energy Production

Seed germination requires a lot of energy and carbon skeleton molecules; as an early event during this process, energy generation is mainly derived from three energy stores, including storage proteins, lipids, and starch [34]. After priming, storage materials in seeds begin to be metabolized, and central carbon metabolism becomes very active. In this study, many DAPs were activated which belonged to sucrose metabolism, glycolysis, pyruvate metabolism, and the TCA cycle (Table 1), and energy supply was thus improved by priming.

Two identified DAPs were involved in the sucrose metabolism pathway in this study. Sucrose synthase (SuSy, A0A0Q3GVZ0), a key enzyme catalyzing the conversion of sucrose into UDP-glucose (UDPG) and fructose [35], was up-accumulated in T24 seeds, indicating that sucrose decomposition was accelerated, thus providing more basic substrates for subsequent glycolysis and energy production. The other DAP, UDP-glucose 6-dehydrogenase (UDP-GlcDH, W5DP16), which was reported to participate in starch and sucrose metabolism and be down-accumulated in deteriorated soybean seeds before harvest [36,37], was up-accumulated in T36 seeds in the present study. The up-accumulation of UDP-GlcDH indicated that sucrose metabolism was promoted by the oxidation catalysis of UDPG to UDP-d-glucuronate. Additionally, UDP-GlcDH was also reported in wheat seeds, where it was involved in ascorbate metabolism to execute function in oxidative responses during artificial aging and priming [33], and its up-accumulation by melatonin priming in this study demonstrated its potential role in resisting oxidative stress caused by aging in oat seeds.

Glycolysis is the metabolic pathway necessary to mobilize storage substances during seed germination, and its key function is to provide energy and material sources (e.g., pyruvate, reductant, and major components for anabolism) for seedling growth and development [38]. Studies have found that the enhanced glycolysis can restrain cell aging by protecting it from oxidative damage caused by ROS, while the inhibition of the glycolytic pathway can lead to cell aging [39]. Melatonin priming in this study induced four DAPs to up-accumulate in glycolysis, namely, ATP-dependent 6-phosphofructokinase (ATP-PFK), glyceraldehyde-3-phosphate dehydrogenase (GAPDH), pyruvate kinase (PK), and S-(hydroxymethyl) glutathione dehydrogenase (S-GSHDH), suggesting that these DAPs might exert positive effects on the germination of aged oat seeds. ATP-PFK (W4ZRX8) is the key enzyme to convert fructose-6-phosphate into fructose-1,6-bisphosphate, which then generates glyceraldehyde 3-phosphate (Gly-3-P) through the subsequent catalysis of fructose-1, 6-diphosphatase [40]. After that, GAPDH (Q6Z9G0) further catalyzes the conversion of Gly-3-P into glycerate 3-phosphate [41]. These two DAPs were up-accumulated by 2.46-fold and 2.09-fold, respectively, after melatonin priming for 24 and 36 h, indicating that melatonin promoted glycolysis and increased C3 metabolites, especially phosphoenolpyruvate (PEP), thus accelerating germination of aged seeds. Meanwhile, PK (A0A1J7GV79), an enzyme that irreversibly converts PEP to pyruvate [42], was significantly increased by 1.72-fold in aged oat seeds after melatonin priming for a duration of 36 h. Based on these above results, the activated ATP-PFK and GAPDH enzymes involved in glycolysis induced the increase in PEP production, and the later period of melatonin priming (T36) efficiently converted PEP to pyruvate owing to the up-regulation of PK, which directly regulated energy production and balance during germination of aged oat seeds.

Pyruvate, the final product of glycolysis, is further converted into acetyl-CoA by the pyruvate dehydrogenase complex, which contains pyruvate dehydrogenase, dihydrolipoyl transacetylase, and dihydrolipoyl dehydrogenase (DLD) [43]. Acetyl-CoA acts as an important intermediate in plant biosynthesis and the TCA cycle. Moreover, pyruvate can be converted into acetaldehyde (HAc), which is then converted to ethanol, through another pathway under the catalysis of pyruvate decarboxylase (PDC) [40]. In this study, the up-accumulated DLD (A0A0D3EN96) and down-accumulated PDC1 (Q9FVE1) in T24 seeds suggested that melatonin promoted the conversion of pyruvate to acetyl-CoA and inhibited its conversion to HAc, thus being able to provide more sufficient and stable substrates for the TCA cycle. Meanwhile, HAc could be subsequently converted into ethanol by a series of reactions owing to the up-accumulation of S-GSHDH (A0A1J3JHF1), so as to achieve biological detoxification. Furthermore, lactoylglutathione lyase, also known as glyoxalase I (GLO1), is a typical detoxifying enzyme of the highly toxic methylglyoxal (MGO), which is produced in glycolysis and reacts with proteins, nucleic acids, and other cellular components [44]. GLO1 (K3Z7G1) was involved in the pyruvate metabolic pathway, and up-accumulated in T36 seeds to obliterate MGO through a two-step detoxification reaction system (i.e., the glyoxalase system). Thus, both S-GSHDH and GLO1 worked together to detoxify and mitigate damage to cells in aged oat seeds.

The TCA cycle is one of the important elements of plant metabolism. It is thought to be responsible for the oxidation of respiratory substrates to drive ATP synthesis and provide energy for cellular activities. As mitochondrial proteins involved in the TCA cycle, aconitate hydratase (ACO) catalyzes the conversion of citrate into isocitrate, and succinate dehydrogenase flavoprotein subunit (SDHA) oxidizes succinate to fumarate and transfers electrons to ubiquinone [45]. In the present study, ACO (Q10S34) was up-accumulated in T12 and T36 seeds, but SDHA (F2E611) was down-accumulated in T12 seeds, with no significant changes in T24 or T36 seeds, revealing that the energy supply for the germination of melatonin-primed seeds mainly relied on the increased level of ACO and not SDHA. Thus, ACO may be the key enzyme that maintains the stability of normal mitochondrial function and provides sufficient energy for cell metabolism in melatonin-primed seeds (T36).

### 3.2. DAPs Involved in Amino Acid Metabolism and Phenylpropanoid Biosynthesis

There were six DAPs identified to associate with amino acid metabolism, including serine hydroxymethyltransferase (SHMT), homoserine dehydrogenase (HSDH), two δ-1-pyrroline-5-carboxylate synthases (P5CSs), phospho-2-dehydro-3-deoxyheptonate aldolase (PDDA), and transaminase/transferase isoform 1 (AT). Additionally, three DAPs were related to phenylpropanoid biosynthesis, namely, phenylalanine ammonia-lyase (PAL), and two peroxidases (PODs) (Table 1). SHMT (A0A0C4BJE5), an enzyme involved in the thymidylate synthase metabolic cycle and catalyzing serine through the retro-aldol cleavage to glycine [46], was up-accumulated by 2.40-fold in T36 seeds in this study, which suggested that glycine synthesis was increased by melatonin priming. *SHMT* had been reported in *Arabidopsis* to play a critical role in regulating abiotic stress-caused damage [47], and in this study, its up-accumulation by melatonin in the protein level indicated that it might be involved in the repairing of oxidative damage that occurred during aging. Additionally, glycine can be synthesized from l-aspartate, via the aspartate-derived amino acid biosynthetic pathway catalyzed by HSDH [48], which was also up-accumulated in T36 seeds 2.58-fold. Yang et al. [49] reported in lettuce (*Lactuca sativa* L.) that appropriate levels of exogenous glycine increased the accumulation of antioxidative compounds (total polyphenols, α-tocopherol) and antioxidant activities, and could be used strategically to improve the nutritional performance of plants. Furthermore, glycine might play a role in melatonin-promoted germination of aged seeds, through the up-regulation of SHMT and HSDH and antioxidant-related effects.

Proline plays an important role in reducing stress-induced damage to plants by removing ROS. It was reported that proline content decreased significantly in aged oat seeds, which consequently led to the loss of vigor [8]. P5CS, an enzyme that catalyzes the first step of proline synthesis, can control proline content by regulating its expression [50]. There were two up-accumulated P5CSs (Q53UC8 and Q43559) in T24 and T36 seeds, demonstrating that melatonin affected proline synthesis, which likely improved seed vigor and germination.

PDDA, an upstream synthetase of amino acids, is involved in the biosynthesis of phenylalanine, tyrosine, and tryptophan, and it is located at the key regulatory point of the synthesis of these three aromatic amino acids [51]. There are two alternative pathways for phenylalanine biosynthesis, and phenylpyruvate aminotransferase (or transaminase, AT) in the alternative phenylpyruvate pathway has been identified in plants to preferentially convert phenylpyruvate to phenylalanine [52]. Phenylalanine can continue to take part in the synthesis of other proteins. According to this study, the accumulation levels of PDDA (A0A1D6RN38) and AT (B6TMW7) were increased in T24 and T36 seeds, indicating that phenylalanine metabolism enhanced by melatonin likely played an important role in renovating the aging-induced damage to seed vigor and germination in oat seeds.

In addition, the generated phenylalanine could be metabolized via the phenylpropanoid pathway, under the essential enzyme PAL to catalyze the non-oxidative degradation of phenylalanine and produce the secondary metabolite *trans*-cinnamic acid (CA) [53]. Then, CA, through a series of reactions, is converted to fumarate in the TCA cycle or to *p*-hydroxy-phenyl lignin (HPL) under the catalysis of POD. Phenylpropanoid is a secondary compound that plays an important role in resisting against abiotic stress [54]. Su et al. [26] found that exogenous melatonin treatment of aged maize seeds induced the up-regulation of differentially expressed genes related to phenylpropanoid biosynthesis. In the present study, PAL (A0A1D6B9G2) and two PODs (W5AX51 and A0A0D3A374) were up-accumulated by 2.74-, 1.70-, and 1.60-fold, respectively, after melatonin priming for 36 h, which suggested that melatonin might activate phenylpropanoid biosynthesis to alleviate the damage caused by aging.

Taken together, the above proteomic results demonstrated that melatonin induced the accumulation of enzymes involved in amino acid metabolism (glycine, serine, proline, and phenylalanine) and phenylpropanoid biosynthesis during the germination of aged oat seeds. These two metabolic processes were closely related to energy generation through intermediate products of pyruvate, PEP, fumarate, and α-ketoglutarate, thus promoting the synthesis of active amino acids and phenylpropanoid. This appears to be the mechanism by which germination-related events were activated by melatonin in aged oat seeds.

### 3.3. DAPs Involved in Phosphatidylinositol Signaling

In eukaryotes, phosphoinositide-specific phospholipase C (PI-PLC) is an omnipresent protein that is involved in the phosphatidylinositol signaling system. It cleaves phosphatidylinositol triphosphate to form diacylglycerol (DAG) and inositol 1,4,5-triphosphate (IP3), which are primary signaling molecules to activate protein kinase C (PKC) and mobilize intracellular Ca^2+^, respectively [55]. Additionally, Ca^2+^ acts as an important messenger to regulate metabolic pathways in many plants under stress [56]. In this study, PI-PLC (I6YMA7) was up-accumulated in T24 and T36 seeds (Table 1), suggesting that phosphatidylinositol signaling was activated by melatonin, thus, facilitating Ca^2+^ to regulate oxidative stress caused by aging.

### 3.4. DAPs Involved in Alpha-Linolenic Acid Metabolism

Phosphatidylcholine (PC), a widespread phospholipid molecule, is an essential structural component that accounts for more than 50% of cell membranes. PC is the most abundant phospholipid, and it plays a crucial role in the maintenance of membrane integrity and function, and multitudinous physiological events, including cell growth, development, energy conversion, and antioxidant defense [57,58]. It contains omega-3 fatty acids, omega-6 unsaturated fatty acids, and linoleic acid, and α-linolenic acid (α-LA) has been found to be a shorter chain omega-3 fatty acid in plants [59]. In addition, α-LA can be indirectly converted into jasmonic acid (JA) through multi-step reactions, under the catalysis of the pivotal enzyme 12-oxo-phytodienoic acid reductase (OPR). It has been reported that multiple isoforms of OPRs occur in diverse annual herbs [60]. The specific OPR identified in this study was up-accumulated by 3.4–4.7-fold in melatonin-primed seeds (T12, T24, and T36), which indicated that melatonin promoted the biosynthesis of JA (Table 1). JA and methyl jasmonate (MeJA), which are widely distributed throughout the plant kingdom, act as signaling molecules in plant oxidative defenses in response to biotic and abiotic stresses [61]. Therefore, the up-accumulation of OPR by melatonin was helpful to promote the synthesis of JA, being further involved in antioxidant defense to maintain intracellular homeostasis, which might be another possible reason for the improvement of germination in aged oat seeds after melatonin priming.

Additionally, melatonin has been demonstrated to act as a plant growth regulator, in a similar way as auxin/indole-3-acetic acid (IAA), to control flowering [62], photosynthesis [63], germination [64], and root morphogenesis [65]. Su et al. [26] found, in aging maize, that melatonin up-regulated the expression of histidine-containing phosphotransfer protein (*AHP*) and a two-component response regulator ARRA family (*ARR-A*), and inhibited the expression of abscisic acid (ABA) and responsive element binding factor (*ABF*), which were involved in cytokinin- and ABA-mediated signaling. It was demonstrated that melatonin played a vital role in regulating hormone signaling transduction. However, different from these growth regulators such as GA, ABA, and cytokinin, another important function of melatonin is that it acts as an antioxidant to decrease ROS, reduce membrane lipid peroxidation, and up-regulate antioxidant enzymes activity in plant stress responses [66,67]. Liang et al. [68] reported in kiwifruit (*Actinidia chinensis* Planch) that melatonin delayed leaf aging through activating the antioxidant capacity and enhancing flavonoid biosynthesis, and there were no hormone-related genes detected. Similarly, in this study, the DAPs involved in hormone signaling that we were interested in at the beginning of the design of this experiment were also not identified. Based on the GO annotation, only ten uncharacterized or predicted proteins were related to hormones among the 3373 detected proteins, but unfortunately, they were all not DAPs under melatonin priming treatments. Meanwhile, in consideration of the results in this study of melatonin priming for 12, 24, and 36 h, there was no significant positive effect on root length and root weight (Figure 1F), suggesting that melatonin might act primarily as an antioxidant rather than a growth regulator.

In conclusion, both this study and the published work proved the same outcome, that melatonin priming improved the germination of aged seeds, whether in a concentration-dependent or time-dependent manner. There were some overlapped pathways found in these two studies, such as energy metabolism (glycolysis, pyruvate metabolism and TCA cycle), protein synthesis, and amino acid metabolism (proline, serine), including DAPs such as SuSy, PK, ACO, P5CS, and SHMT [9]. However, the mechanisms involved in the time-course dependent manner of melatonin priming still differed from those identified in the process relaying on its concentrations [9]. Based on the thorough analysis, three main differences were revealed. (a) Cellular structures were repaired in different ways. In time-dependent priming, melatonin might restore membrane integrity by the improved antioxidant capacity, which was affected by JA synthesis due to the increased OPR level, while in concentration-dependent priming, melatonin repaired ultrastructures through inhibiting phospholipase D (PLD) abundance and phospholipid degradation. (b) Energy production was different. Glycolysis and the TCA cycle seemed to be the main energy generation pathways due to high levels of GAPDH, PK, and DLD in time-dependent priming, but glycolysis and β-oxidation of lipids served as energy sources owing to the accumulation of phosphoenolpyruvate carboxylase 2 (PEPCase2) and 3-ketoacyl-CoA thiolase-like protein (KATLP) in concentration-dependent priming. (c) Phenylpropanoid biosynthesis was activated by melatonin to regulate oxidative stress in time-dependent priming, which was not involved in the concentration-dependent process.

## 4. Materials and Methods

### 4.1. Seed Materials

Oat (*Avena sativa* L. cv. ‘Cayuse’) seeds were obtained from Rytway Ecotechnology Company (Beijing, China), with an original GP of 100% and moisture content (MC) of 8.9% (fresh basis). Upon reception, seeds were selected, dehulled, and adjusted to 10% MC. Then, seeds were immediately sealed in aluminum foil bags (120 × 170 mm, approx. 25 g in each bag) and stored at −20 °C in the dark prior to further use. The whole workflow of this experiment is shown in Figure S1.

### 4.2. Seed Aging and Melatonin Priming Treatments

Oat seeds were incubated at 45 °C for 48 days to obtain aged seeds with 70% GP [9]. A single layer of aged seeds, with embryos tightly attached to the filter paper in a Petri dish (110 × 110 mm), were primed with 10 μM of melatonin (determined according to the screening results of preliminary tests; Figure S2), and with 15 mL of solution to immerse embryos, at 20 °C for 12, 24, and 36 h in the dark. Then, seeds were washed with distilled water three times, surface-dried with filter paper, and air-dried back to 10% MC at 20 °C and 33% relative humidity. Afterwards, seeds were used for further analysis, including analyses of germinability and ultrastructural, physiological, and proteomic changes. Two groups of seeds were prepared: (a) aged seeds (marked as CK); and (b) melatonin-primed seeds, namely the aged seeds that were primed with melatonin for various durations (T12, T24, and T36 treatments, respectively).

The embryos were extracted with a scalpel on ice, after seed imbibition (15 mL of distilled water) at 20 °C for 12 h in the dark. For ultrastructural observation, the isolated embryos were fixed in glutaraldehyde solution; for physiological analysis, protein extraction, and qRT-PCR assays, the isolated embryos were immediately frozen in liquid nitrogen, and then stored at −80 °C.

### 4.3. Quantification of Endogenous Melatonin

Melatonin was extracted using the method described by Pothinuch and Tongchitpakdee [69]. Embryo samples (0.2 g) were pulverized with liquid nitrogen and homogenized in 5 mL of methanol. The homogenates were centrifuged at 10,000× *g* for 15 min at 4 °C, after ultrasonication (80 Hz) at 45 °C for 40 min. The extracts were dissolved in 1 mL of 5% methanol and purified using a C18 solid phase extraction cartridge (Waters). The sample solution was eluted through a 0.1 μm syringe filter by 1 mL of 80% methanol, and then assayed by UHPLC-ESI-MS/MS (UHPLC-1290 Series and a 6460 QqQ-MS/MS; Agilent Technologies). The excitation and emission wavelengths were at 285 and 345 nm, respectively. Melatonin content was calculated by comparing the peak area (% fluorescence) of the sample with that of its standard curve.

### 4.4. Germination Test and Seedling Growth Assay

Germination assays were conducted on the basis of the criteria in ISTA Rules chapter V [70]. Four replicates of 50 seeds each were placed into Petri dishes, with three layers of filter papers that were dampened using 10 mL of distilled water. Then, seeds were incubated in a germination chamber under a constant temperature of 20 °C, with 8 h of light and 16 h of dark. Germination was checked daily for 10 days, and the normal seedlings without lesions or morphological defects were recorded. On the 10th day, all normal seedlings were taken out, and their SL and RL, SW and RW (fresh basis) were measured [71]. The GP, VI, GI, and SVI were calculated.

### 4.5. Ultrastructural Observation of Radicle Cells

The isolated imbibed embryos were randomly selected, and radicles were cut into sections transversely and fixed into 2 mL of 4% glutaraldehyde solution for 48 h, before being placed in a refrigerator at 4 °C. The other preparation procedures for transmission electron microscopy were conducted according to the method of Yan et al. [11].

### 4.6. Determination of H_2_O_2_ and MDA Contents

The H_2_O_2_ content was measured using an H_2_O_2_ Assay Kit (Nanjing Jianchen Bioengineering Institute, Nanjing, China), according to the manufacturer’s instruction. The MDA content was determined according to the method of Bailly et al. [72], which was calculated by measuring absorbance values at 532 and 600 nm. The isolated embryos (0.2 g) were ground into powder and extracted with 5% (w/v) trichloroacetic acid, before being centrifuged at 15,000× *g* for 20 min at 4 °C. Then, 2.5 mL of extracting solution was mixed into 2.5 mL of a reaction system that contained 0.5% thiobarbituric acid and 5% (*w/v*) trichloroacetic acid.

### 4.7. Assay of Antioxidant Enzymes

Antioxidant enzymes were extracted according to our previously described methods [9]. The SOD (EC 1.15.1.1) activity was assayed based on the method of Beauchamp and Fridovich [73] as described by Dhindsa and Matowe [74], which measured the inhibition of photochemical reduction of nitroblue tetrazolium at 560 nm. One enzyme unit referred to the volume of supernatant corresponding to 50% inhibition of this reaction. CAT (EC 1.11.1.6) activity was measured by the dynamic change of absorbance value at 240 nm within one minute, due to the decline of H_2_O_2_ extinction [75]. MDHAR (EC1.6.5.4) activity was determined by the decrease in absorbance at 340 nm because of NADH oxidation [76]. DHAR (EC 1.8.5.1) activity was assayed by measuring the increase in absorbance at 265 nm as a result of ascorbic acid formation [77]. APX (EC 1.11.1.11) activity was determined by monitoring the decrease in absorbance at 290 nm within one minute, owing to ascorbic acid oxidation [78]. GR (EC 1.6.4.2) activity was measured by the decrease in absorbance at 340 nm, caused by NADPH oxidation [79].

### 4.8. Extraction and Quantification of Embryo Proteins

The already isolated embryos (stored at −80 °C) were used to extract proteins, with two biological replicates (200 per biological sample, determined based on the principle of tolerance for germination in ISTA Rules chapter V [70]) for each treatment (CK, T12, T24, and T36). Embryos were pulverized using liquid nitrogen, mixed with 200 μL of ice-cold lysis buffer (50 mM Tris-HCl, pH 8.0, 8 M Urea, 2 M Thiourea, 0.1% SDS), and then suspended by an ultrasonic processor for 15 min. After centrifugation at 13,000× *g* rpm for 20 min at 4 °C, the supernatant was mixed well with 800 μL of ice-cold acetone (containing 10 mM DTT) and incubated for approximately two hours, before being centrifuged again. The collected precipitate was resuspended with ice-cold acetone, and centrifuged at 13,000× *g* rpm for 20 min at 4 °C. The protein pellet was air-dried and resuspended in 100 μL of lysis buffer. The protein concentration was determined by the Bradford assay.

### 4.9. Protein Reduction, Digestion, and iTRAQ Labeling

Approximately 100 µg of proteins from each sample were mixed with 10 mM DTT and incubated at 37 °C for one hour. After reduction and alkylation by 55 mM iodoacetamide at room temperature for one hour in the dark, proteins were digested with 2 μg of 50:1 trypsin (Promega, Madison, WI, USA) at 37 °C for 12 h before centrifugation at 12,000× *g* for 15 min. Digested proteins were then acidulated with an equal volume of 0.1% formic acid, purified with a Strata-X C18 column (8B-S100-UBJ; Phenomenex, Torrance, CA, USA) three times, washed twice with 0.1% formic acid and 5% acetonitrile, and eluted with 0.1% formic acid and 80% acetonitrile. The peptides were dried in a vacuum, and then reconstituted in 0.5 M TEAB solution (pH 8.5).

Proteins were labeled using an 8-plex iTRAQ Reagent Multiplex Kit (AB Sciex, Foster City, CA, USA) according to the manufacturer’s instruction. The aged control group was labeled with 113- and 114-isobaric tags, and the melatonin-primed experimental group was labeled as follows: 115- and 116- isobaric tags for T12, 117- and 118- isobaric tags for T24, and 119- and 121- isobaric tags for T36. The labeled samples were incubated at 25 °C for one hour, before being stopped with 100 μL of ddH_2_O. Afterwards, the differentially labeled peptide mixtures were pooled and dried by centrifugation in a vacuum.

### 4.10. NanoLC-MS/MS Analysis

Labeled peptide samples were re-dissolved in 100 μL of mobile phase A (2% acetonitrile, 20 mM NH_4_FA, pH 10.0, adjusted with NH_3_·H_2_O), and classified using a high-performance liquid chromatography (HPLC) system (Thermo Dionex Ultimate 3000 BioRS; Thermo Fisher Scientific, Waltham, MA, USA) with a Durashell C18 column (4.6 × 250 mm, 5 μm, 100 Å; Agela, Wilmington, DE, USA). Peptides were separated using a gradient elution of 5% mobile phase B (80% acetonitrile, 20 mM NH_4_FA, pH 10.0, adjusted with NH_3_·H_2_O) for 7 min, 25% B for 16 min, and 5% B for 25 min. The flow rate of elution was 1 mL/min, and the absorbance at 214 nm was measured. A total of 12 fractions were collected every minute, desalted with a C18 column (Strata-X, Phenomenex), and then dried by vacuum centrifugation.

Fractions were re-dissolved in mobile phase A (0.1% formic acid, 5% acetonitrile), and separated using a nano LC-MS/MS system connected to a Q-exactive HF-X mass spectrometer (Thermo Fisher Scientific). Peptides were loaded onto the Eksigent Chromxp Trap Column (C18-CL, 350 μm × 0.5 mm, 3 μm, 120 Å, AB Sciex) using an autosampler, with a flow rate of 10 μL/min for 5 min, and then eluted onto an analytical C18 column (75 μm inner diameter, 10 cm length, 3 μm, AB Sciex). The samples were eluted with a gradient of mobile phase B (0.1% formic acid, 95% acetonitrile) as follows: 5%–30% B for 0–65 min; 30%–50% B for 65–70 min; 50%–80% B for 70–85 min; and 80%–5% B for 85–90 min, with a 300 nL/min flow rate.

The MS/MS analysis was conducted with a mass spectrometer in the data-dependent mode. Data were acquired using an ion source gas 1 of 5 psi, curtain gas of 35 psi, ion spray voltage floating of 2.5 kV, and interface heater temperature of 150 °C. The MS1 scan spectra (350–1500 *m/z*) were collected for 250 milliseconds, and mass tolerance was 50 mDa; the MS2 spectra (100–1500 *m/z*) were collected for 100 milliseconds; and dynamic exclusion was set to 12 s.

### 4.11. Database Search, Bioinformatic Analysis, Protein Annotation, and Functional Analysis

Raw data were analyzed using Proteome Discoverer 2.1 (Thermo Fisher Scientific) against all plant proteins in the Uniprot protein database (downloaded on December 20, 2017; 2,304,711 protein sequences). Parameters for protein identification were as follows: the mass tolerance was set to 10 ppm for the precursor ion and 0.02 Da for the fragment ion; the maximum missed cleavages for trypsin digestion were set to two. Carbamidomethyl (C) and iTRAQ8plex (N-terminal, K) were specified as fixed modifications, and oxidation (M) and acetyl (N-terminal) were specified as dynamic modifications. In order to reduce the number of false positive identifications and improve the quality of analysis results, due to the limitations of two biological replicates, peptides with a false discovery rate (FDR) of 1% and at least one unique peptide were selected for further analysis. The abundance of all markers in each peptide spectra match (PSM) was first calculated, and then the abundance of all unique spectra contained in each protein was summed to quantify protein abundance, which was further normalized. The mix of two iTRAQ experiments was used as a bridge to normalize the results, and the ratio was calculated. To determine the significance of difference, *t*-tests were conducted, and the *p*-value was calculated. The identified proteins with an FC ≥ 1.5 (or ≤0.67) and *p* ≤ 0.05 were considered as DAPs between aged seeds and melatonin-primed seeds (i.e., T12 vs. CK, T24 vs. CK, and T36 vs. CK).

The DAPs were assessed according to their Cluster of Orthologous Groups (COG) category and annotation. Gene ontology (GO) annotation, including biological process, cellular component, and molecular function, was determined using the Uniprot database (http://www.uniprot.org/). The Kyoto Encyclopedia of Genes and Genomes (KEGG) database (http://www.genome.jp/kegg/pathway.html) was used to predict the main metabolic pathways.

### 4.12. RNA Extraction and qRT-PCR

Total RNA from oat embryos was extracted using a TRNzol Kit (Tiangen Biotech, Beijing, China), according to the manufacturer’s instructions. Reverse transcription was performed using a PrimeScript™ RT reagent kit with gDNA Eraser (Takara, Dalian, China). Gene expression was detected by qRT-PCR using SYBR^®^ Premix Ex Taq™ II (Tli RNaseH Plus) (Takara, Dalian, China), following the manufacturer’s instructions, with the ABI7900 Real-Time PCR thermal cycler (Applied Biosystems, Waltham, MA, USA). The *ACTIN2* reference gene was used to normalize the relative expression levels of candidate genes, which were calculated with the 2^−∆∆*C*t^ method. All primer sequences are listed in Table 2.

### 4.13. Statistical Analysis

Results are presented as mean values with standard errors. One-way analysis of variance (ANOVA) was conducted, followed by Duncan’s test, using SPSS Statistics software (version 17.0, SPSS Inc., Chicago, IL, USA). Differences were considered to be statistically significant at a *p* < 0.05 threshold.

## 5. Conclusions

**Figure 9:**
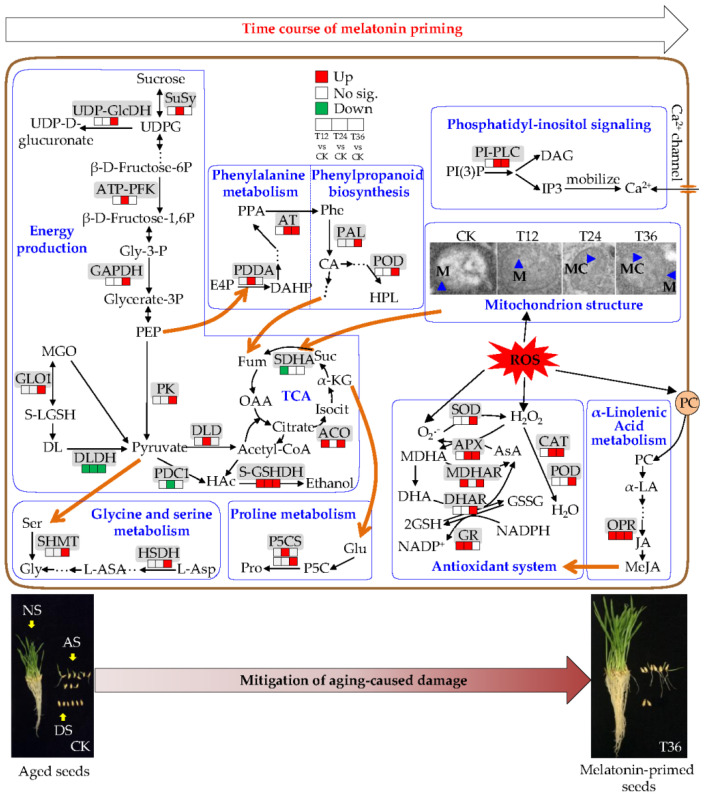
Putative pathways for time-course physiological and proteomic mechanisms underlying melatonin priming on renovating aging-induced damage and promoting germination in oat seeds. SuSy, sucrose synthase; UDP-GlcDH, UDP-glucose 6-dehydrogenase; UDPG, UDP-glucose; ATP-PFK, ATP-dependent 6-phosphofructokinase; Gly-3-P, Glyceraldehyde-3P; GAPDH, glyceraldehyde-3-phosphate dehydrogenase; PEP, phosphoenolpyruvate; PK, pyruvate kinase; MGO, methylglyoxal; GLO1, lactoylglutathione lyase; S-LGSH, (R)-S-lactoylglutathione; DL, D-lactate; DLDH, D-lactate dehydrogenase [cytochrome]; DLD, dihydrolipoyl dehydrogenase; PDC1, pyruvate decarboxylase 1; HAc, acetaldehyde; S-GSHDH, S-(hydroxymethyl) glutathione dehydrogenase; OAA, oxaloacetate; ACO, aconitate hydratase; Isocit, Isocitrate; α-KG, α-ketoglutarate; Suc, succinate; SDHA, succinate dehydrogenase [ubiquinone] flavoprotein subunit; Fum, fumarate; Ser, serine; SHMT, serine hydroxymethyltransferase; Gly, glycine; HSDH, homoserine dehydrogenase; L-ASA, L-aspartate 4-semialdehyde; L-Asp, L-Aspartate; Pro, proline; P5CS, δ-1-pyrroline-5-carboxylate synthase; Glu, glutamate; E4P, D-erythrose 4-phosphate; PDDA, phospho-2-dehydro-3-deoxyheptonate aldolase; DAHP, 3-deoxy-D-arabino-heptulosonate-7-phosphate; PPA, phenylpyruvate; AT, transaminase; Phe, phenylalanine; PAL, phenylalanine ammonia-lyase; CA, cinnamic acid; HPL, p-hydroxy-phenyl lignin; POD, peroxidase; PI(3)P, phosphatidylinositol triphosphate; PI-PLC, phosphoinositide phospholipase C; DAG, diacylglycerol; PC, phosphatidylcholine; α-LA, α-linolenic acid; OPR, 12-oxo-phytodienoic acid reductase; JA, jasmonate; MeJA, methyl jasmonate.

The comprehensive analyses of germinability and seedling growth phenotype, cellular ultrastructure, and physiological changes, indicated that melatonin priming restored damage associated with aging in a time-course dependent manner, and the T36 treatment had the greatest potential and best remediation effect of the three treatments tested. Based on the proteomic analysis of aged and melatonin-primed seeds, the possible mechanisms and a putative model for melatonin promoting germination were developed (Figure 9). Thus, two primary findings were revealed in this study. Firstly, melatonin might restore membrane integrity by increasing level of OPR to promote the synthesis of JA, which further contributed to improving the antioxidant capacity of the AsA–GSH cycle and also participated in oxidative defense; therefore, cellular ultrastructures could be repaired. Secondly, the relatively intact cell structures enabled the metabolism of glycine, serine, proline, and phenylalanine, and the biosynthesis of phenylpropanoid, which were closely related to energy generation through the intermediate products of pyruvate, PEP, fumarate, and α-ketoglutarate, thus providing energy, active amino acids, and secondary metabolites for germination to be restored in aged seeds. The present study helped us to understand the pathways and mechanisms underlying the time-course dependent responses of aged oat seeds to melatonin priming.

## Figures and Tables

**Table 1:** DAPs that significantly altered under time course of melatonin priming in aged oat seeds; FC, fold change.

Accession	Description	T12 vs. CK		T24 vs. CK		T36 vs. CK	
		FC	*p*-Value	FC	*p*-Value	FC	*p*-Value
**Carbon Metabolism and Energy Production**							
**Starch and Sucrose Metabolism**							
A0A0Q3GVZ0	Sucrose synthase	1.200	0.0094	1.509	0.0001	1.309	0.0074
W5DP16	UDP-glucose 6-dehydrogenase	1.182	0.2406	0.902	0.4743	1.523	0.0294
Glycolysis/Gluconeogenesis							
W4ZRX8	ATP-dependent 6-phosphofructokinase	1.307	0.2254	2.459	0.0112	1.682	0.0598
Q6Z9G0	Glyceraldehyde-3-phosphate dehydrogenase	1.160	0.7688	1.683	0.0828	2.091	0.0355
A0A1J7GV79	Pyruvate kinase	1.367	0.0435	1.398	0.0200	1.722	0.0055
A0A1J3JHF1	S-(hydroxymethyl)glutathione dehydrogenase (Fragment)	2.110	0.0164	1.998	0.0101	1.837	0.0157
Pyruvate metabolism							
A0A0D3EN96	Dihydrolipoyl dehydrogenase	1.288	0.1383	1.551	0.0333	1.475	0.0628
Q9FVE1	Pyruvate decarboxylase 1 (Fragment)	0.733	0.0275	0.659	0.0010	0.682	0.0053
K3Z7G1	Lactoylglutathione lyase	1.224	0.0194	1.297	0.0029	1.653	0.0041
A0A1J3DH40	D-lactate dehydrogenase [cytochrome], mitochondrial (Fragment)	0.234	0.0023	0.535	0.0096	0.618	0.0294
Citrate cycle (TCA cycle)							
Q10S34	Aconitate hydratase	1.680	0.0076	1.206	0.1089	1.602	0.0193
F2E611	Succinate dehydrogenase [ubiquinone] flavoprotein subunit, mitochondrial	0.520	0.0072	0.818	0.0199	1.004	0.9945
**Amino acid metabolism**							
Phenylalanine, tyrosine and tryptophan biosynthesis							
A0A1D6RN38	Phospho-2-dehydro-3-deoxyheptonate aldolase	0.863	0.1420	2.406	0.0033	1.656	0.0617
B6TMW7	Transaminase/ transferase isoform 1	1.716	0.0775	2.051	0.0366	2.062	0.0479
Phenylalanine metabolism							
A0A1D6B9G2	Phenylalanine ammonia-lyase	0.628	0.1998	1.336	0.0155	2.739	0.0393
Arginine and proline metabolism							
Q43559	Delta-1-pyrroline-5-carboxylate synthase	1.455	0.2157	3.981	0.0004	1.970	0.1216
Q53UC8	Delta-1-pyrroline-5-carboxylate synthase	1.405	0.0044	1.334	0.0282	1.608	0.0024
Glycine, serine and threonine metabolism							
A0A0C4BJE5	Serine hydroxymethyltransferase	1.574	0.1904	1.473	0.2255	2.576	0.0203
A0A0D3HHP5	Homoserine dehydrogenase	1.177	0.8198	1.722	0.1573	2.398	0.0495
**Phenylpropanoid biosynthesis**							
W5AX51	Peroxidase	1.457	NA	1.362	0.0445	1.696	0.0441
A0A0D3A374	Peroxidase	1.399	0.0117	1.358	0.0206	1.604	0.0006
A0A1D6B9G2	Phenylalanine ammonia-lyase	0.628	0.1998	1.336	0.0155	2.739	0.0393
**Phosphatidylinositol signaling**							
I6YMA7	Phosphoinositide phospholipase C	1.208	0.0467	1.704	0.0002	1.548	0.0023
**alpha-linolenic acid metabolism**							
H9CWE9	12-oxo-phytodienoic acid reductase	3.499	0.0330	4.034	0.0355	4.771	0.0185

**Table 2:** Primer sequences of qRT-PCR in this study.

Accession	Primer F (5′-3′)	Primer R (5′-3′)
H9CWE9	GACCACGGCATCCTCTACC	CTTGGGCAGGTCTGGGTT
A0A078HA44	CAGCGTCATTGGTACATCCAG	TTTCATCAAGCTCACGGCAC
A0A1J3DH40	CTTGTGGGCTTGCTTTGC	TTCGCTGCTCTTCGTTGC
Q2R2B4	ATGATGAACTGGTGCCTGGT	GCCATTGCCATTGCTCTT
*ACTIN2*	GATGATGCGCCAAGAGCTG	GCCTCATCACCTACGTAGGCAT

## Data Availability

The data supporting the results of this article are included in the main text and Supplementary Materials. The proteomics raw data were deposited to iProX database (project ID: IPX0002675000), and downloaded through the access link https://www.iprox.org/page/project.html?id=IPX0002675000. Additionally, we had already finished this work when the available OT3098 oat genomes were published in November 2019; therefore, there might be some deviations in the results of proteomic analysis based on all plant proteins.

## Supplementary Materials

Supplementary materials can be found at https://www.mdpi.com/1422-0067/22/2/811/s1.

## Abbreviations

ACO	Aconitate hydratase
APX	Ascorbate peroxidase
AsA–GSH	Ascorbate–glutathione
ATP-PFK	ATP-dependent 6-phosphofructokinase
CAT	Catalase
DHAR	Dehydroascorbate reductase
DLD	Dihydrolipoyl dehydrogenase
DLDH	D-lactate dehydrogenase
GAPDH	Glyceraldehyde-3-phosphate dehydrogenase
H_2_O_2_	Hydrogen peroxide
HSDH	Homoserine dehydrogenase
iTRAQ	Isobaric tags for relative and absolute quantification
MDA	Malondialdehyde
MDHAR	Monodehydroascorbate reductase
OPR	12-oxo-phytodienoic acid reductase
PAL	Phenylalanine ammonia-lyase
P5CS	δ-1-pyrroline-5-carboxylate synthase
PDC1	Pyruvate decarboxylase 1
PDDA	Phospho-2-dehydro-3-deoxyheptonate aldolase
PI-PLC	Phosphoinositide phospholipase C
PK	Pyruvate kinase
POD	Peroxidase
ROS	Reactive oxygen species
SHMT	Serine hydroxymethyltransferase
SOD	Superoxide dismutase
SuSy	Sucrose synthase
UDP-GlcDH	UDP-glucose 6-dehydrogenase
