# Supplementary material for: Comparative Time-Course Physiological Responses and Proteomic Analysis of Melatonin Priming on Promoting Germination in Aged Oat (Avena sativa L.) Seeds
Source: Int J Mol Sci. 2021 Jan 15;22(2):811. doi: 10.3390/ijms22020811 (PMC7830126; doi:10.3390/ijms22020811)
Supplement: Supplementary file 1 [file ijms-22-00811-s001.zip › ijms-1059791-supplementary/Supplementary Files/Figure S2.docx]

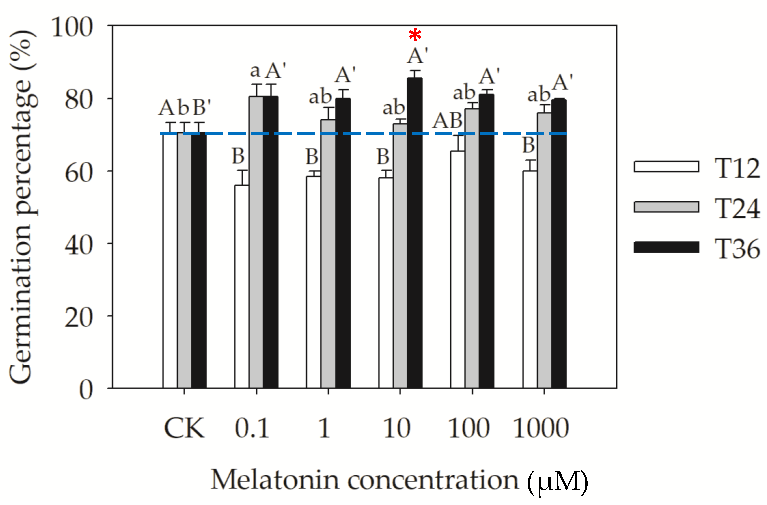


**Figure S2.** Changes of germination percentage of aged oat seeds after melatonin priming treatments. The aged seeds were primed with different concentrations of melatonin (0.1, 1, 10, 100, and 1000 μM, with 15 mL of solutions to immerse embryos) at 20 °C for 12, 24, and 36 h in the dark (T12, T24, and T36). Values represent the means ± SE from four replicates. One-way ANOVA was adopted to perform the statistical analysis. Different capital letters (e.g. A, B), lowercase letters (e.g. a, b), and capital letters plus apostrophe (e.g. A’, B’), respectively, indicated significant differences among different concentrations of melatonin at T12, T24, and T36.
