# Supplementary figures and images for: Comparative Time-Course Physiological Responses and Proteomic Analysis of Melatonin Priming on Promoting Germination in Aged Oat (Avena sativa L.) Seeds
Source: Int J Mol Sci. 2021 Jan 15;22(2):811. doi: 10.3390/ijms22020811 (PMC7830126; doi:10.3390/ijms22020811)

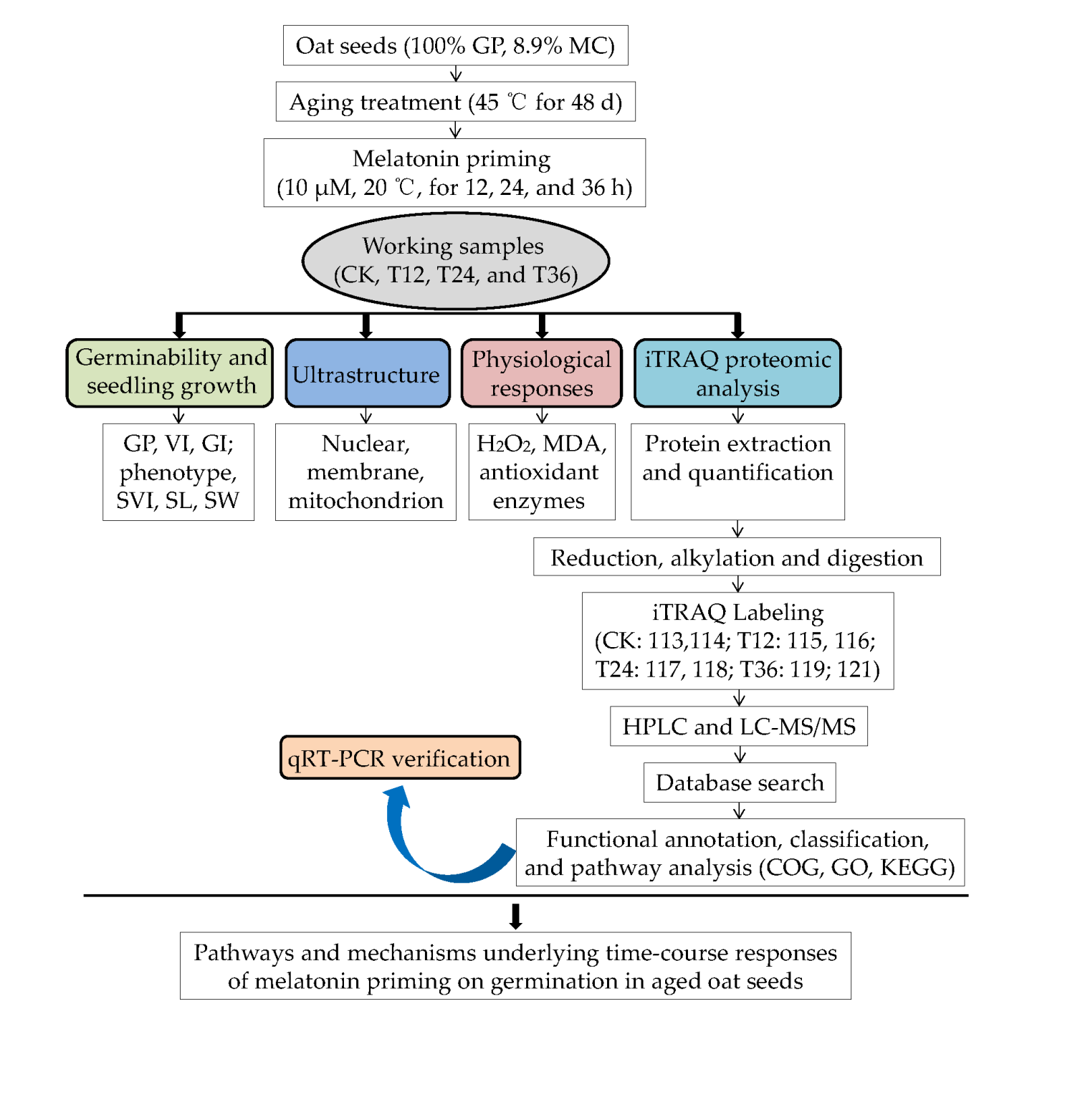


**Figure S1.** Workflow for the whole study.

Supplement: Supplementary file 1 [file ijms-22-00811-s001.zip › ijms-1059791-supplementary/Supplementary Files/Figure S1.docx]
